# Cutaneous Phaeohyphomycosis of the Right Hand Caused by *Exophiala jeanselmei*: A Case Report and Literature Review

**DOI:** 10.1007/s11046-022-00623-y

**Published:** 2022-03-21

**Authors:** Chongyang Wu, Ling Shu, Zhixing Chen, Qianrong Hu, Lijun Tao, Chao He

**Affiliations:** 1 Department of Laboratory Medicine, West China Hospital of Sichuan University, Chengdu, 610041 Sichuan China; 2 West China School of Medicine, Sichuan University, Chengdu, 610041 China; 3 The First Hospital of Kunming, Kunming, 650011 Yunnan China

**Keywords:** Phaeohyphomycosis, *Exophiala jeanselmei*, Infection, Literature review

## Abstract

*Exophiala* spp. is increasingly reported as a pathogen causing the cutaneous, subcutaneous or invasive infection. In this report, we present a case of cutaneous phaeohyphomycosis due to *E. jeanselmei* on the right hand of a farmer*,* who suffered from this disease three years ago which had not been definitely diagnosed until he was admitted to our hospital. In our hospital, a potential fungal pathogen was observed by histopathological examination, and then was recovered and identified as *E. jeanselmei* by sequencing its internal transcribed spacer region. After 4 weeks of antifungal treatment, his hand recovered very well. To investigate the in vitro susceptibility of *E. jeanselmei* isolates to antifungal agents and compare the characteristics of their related infections among immunocompetent and immunocompromised patients, we reviewed 84 cases published in PubMed database between 1980 and 2020.

## Introduction

Phaeohyphomycosis is a group of rare fungal infections caused by the dematiaceous fungi, such as *Alternaria* spp. *Phialophora* spp. and *Exophiala* spp. [1]. Recently, *Exophiala* spp. has been frequently reported as an etiologic agent of phaeohyphomycosis in both immunocompromised and immunocompetent individuals [2–5], which highlights the importance of reviewing the characteristics of this pathogen related infections. In this study, we described one case of prolonged cutaneous infection due to *E. jeanselmei* and reviewed 84 cases published in PubMed database during 1980 to 2020.

## Case Report

**Fig. 1:**
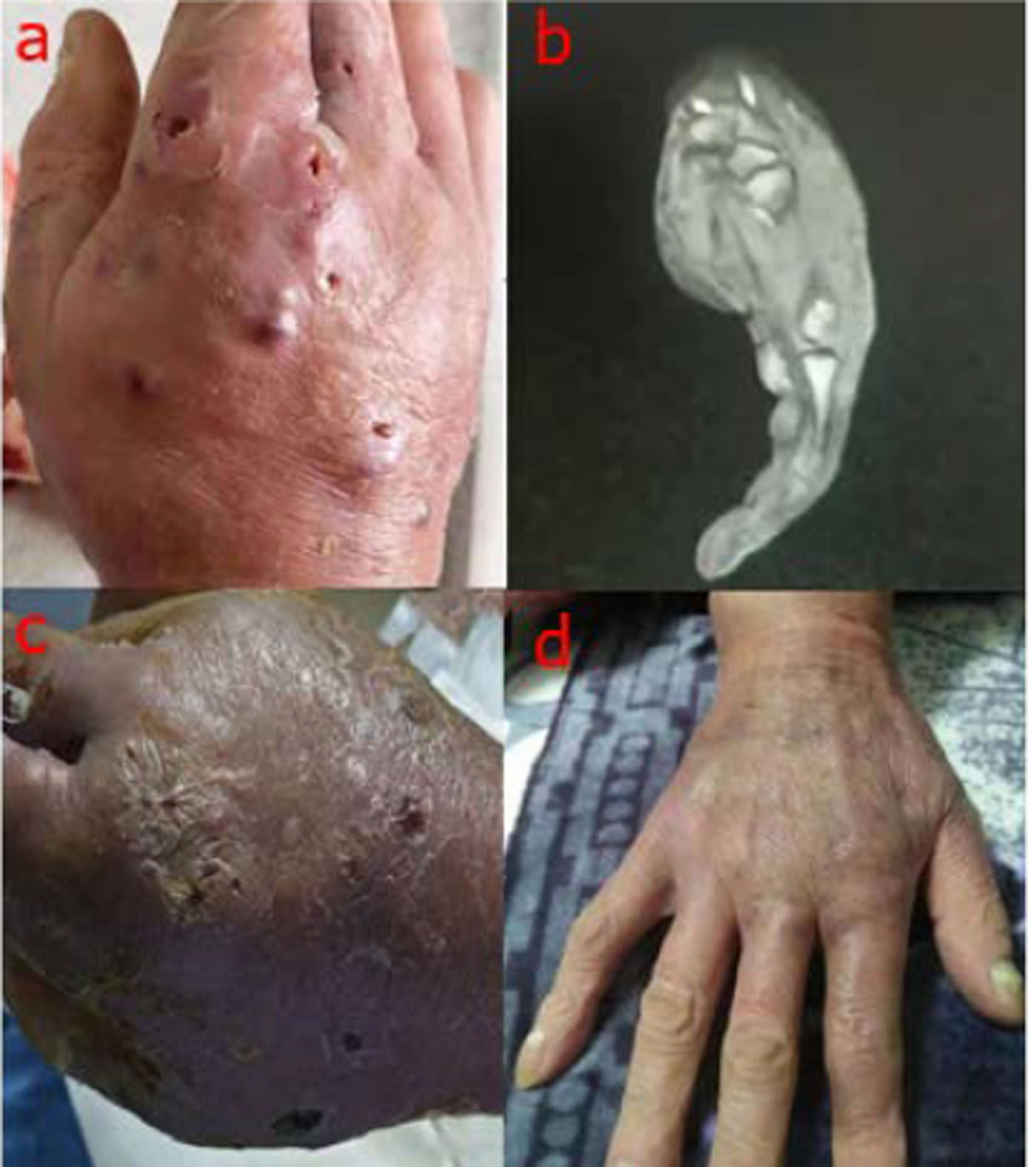
The presentation of the right hand before and after antifungal treatment in our case. **a** There were multiple cutaneous abscesses on the back of the red, swollen and painful right hand when the patient was admitted to our hospital. **b** Computed tomography scan of the hand showed the extensive tissue swelling in the wrist and palm. **c** After one week treatment with amphotericin B, the pus no longer discharged and the skin began to scab. **d** Voriconazole was administered for the patient after the isolate was identified as *E. jeanselmei*, the hand recovered very well after another two weeks

A 61-year-old male farmer was admitted to West China Hospital of Sichuan University on October 30, 2018. There were multiple cutaneous abscesses on the back of the red, swollen and painful right hand (Fig. 1a). Computed tomography (CT) scan of this hand showed the extensive tissue swelling in the wrist and palm (Fig. 1b).

He complained that this hand was injured when he worked in the field three years ago. And then the swelling and pain was developed, while the definite diagnosis was not made in the local hospital and triamcinolone acetonide was administrated intermittently to relieve the discomfort of this hand. Two months ago, the old skin lesions in this hand expanded rapidly and new ones appeared. Some kinds of the antibiotics were administered in the local hospital but the symptoms did not improve. He denied the history of autoimmune diseases, diabetes and tumors.

**Fig. 2:**
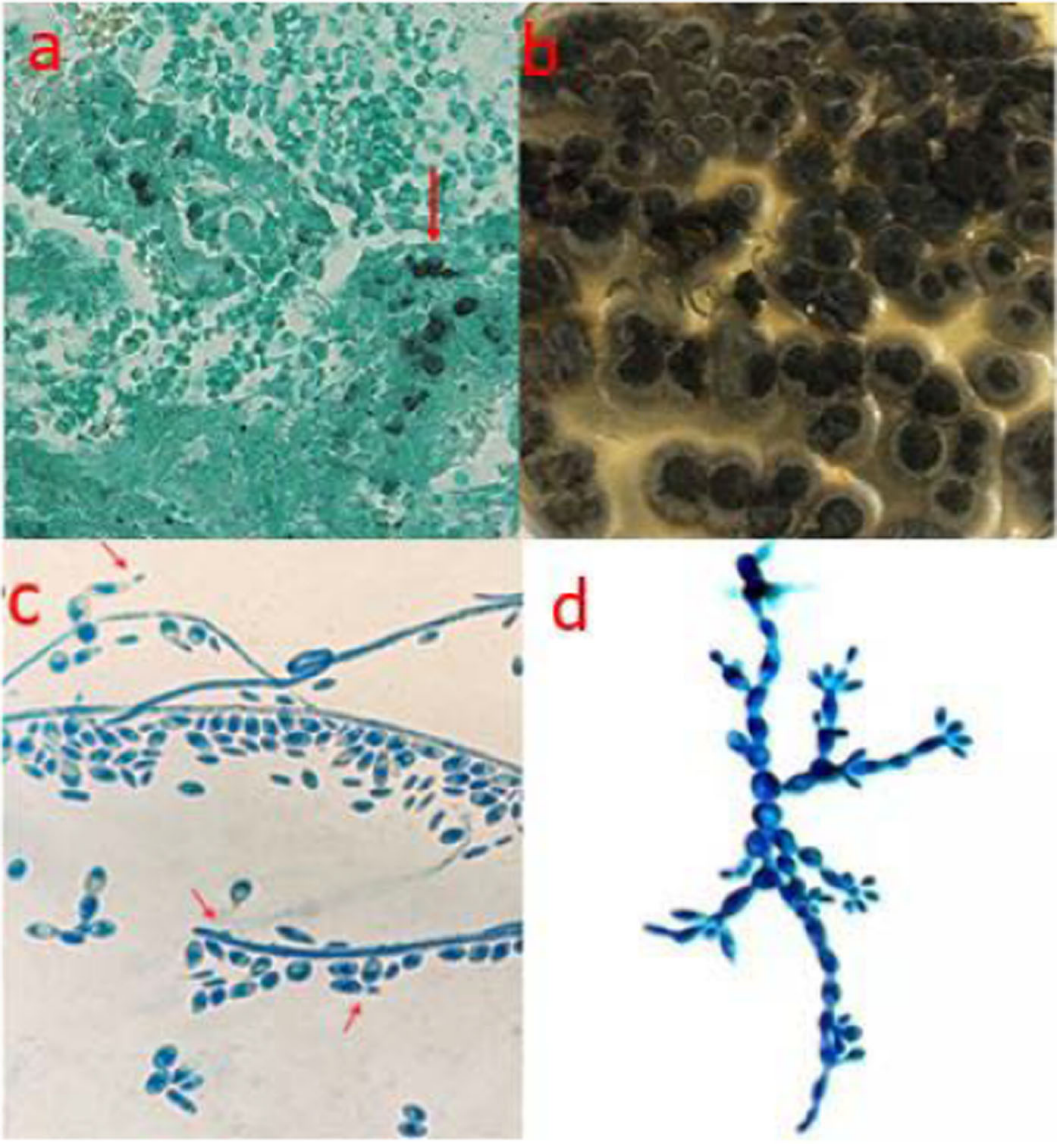
The positive results of the specimens for pathogenic examination in our case. **a** Multiple dark hyphae were highlighted with Gomori methenamine silver (GMS) staining of skin biopsy as the arrows point to. **b** Flat to domed, mucoid, dark olive-green to black colonies with black reverse growed at 28 °C on Sabouraud’s dextrose agar (SDA) after one week incubation. **c** The septate hyphae, conidia, and annellides ware stained with Lactophenol cotton blue for the isolate incubated on SDA plate for one week. **d** The septate hyphae, conidia, and annellides ware stained with Lactophenol cotton blue for the isolate incubated on Potato dextrose agar (PDA) plate for three weeks

At his admission to our hospital, white blood cell count (7.27 × 10^9^/L), neutrophilic granulocyte ratio (85.3%), serum C-reactive protein (20.76 mg/L) and procalcitonin (0.22 ng/mL) were all elevated. The skin biospy of this hand was sent for pathological investigation. Multiple dark hyphae were highlighted with Gomori methenamine silver (GMS) staining (Fig. 2a).

Thick brown aspirate collected by fine-needle was sent for bacterial, fungal, and mycobacterial investigation. The results of Gram staining and acid-fast staining were negative. There were flat to domed, mucoid, dark olive-green to black colonies with black reverse at 28 °C on sabouraud’s dextrose agar (SDA) plate (Fig. 2b). The colonies were stained with lactophenol cotton blue. The septate hyphae, single-celled and ellipsoidal conidia accumulating in groups, and cylindrical to flask-shaped annellides with narrow apical area, were observed (Fig. 2c, 2d).

Isolation of *Exophiala* spp. from the aspirate was reported to the clinicians. This patient was administered with amphotericin B (1 mg/kg iv q12h) immediately. After one week, the pus no longer discharged and the skin began to scab (Fig. 1c).

Sequencing the internal transcribed spacer (ITS) was performed to identify the isolate to species level as previously reported [6], and its sequence were completely homology with those of *E. jeanselmei* D12H (Accession ID: MH010928.1).

In vitro susceptibility testing of this isolate to antifungal agents was performed according to the M38-A2 protocol of Clinical and Laboratory Standards Institute (CLSI) [7]. Briefly, the conidia was collected with a cotton swab and suspended in sterile saline with Tween. Heavy particles were settled for 3 to 5 min. The supernatant was collected and mixed with a vortex mixer. Its turbidity was adjusted to a 0.5 McFarland Standard. And then, 100 μL of the suspension was added to 11 mL of Sensititre YeastOne broth (Thermo Fisher Co.) to give a final inoculum. The final suspension was transferred into the Sensititre YeastOne plate and incubated at 35 °C in a non-CO_2_ incubator. The minimum inhibitory concentrations (MICs) were reported as listed in Table 1. Voriconazole (200 mg iv q12h) was administered for this patient. One week later, he was discharged with oral voriconazole (200 mg, bid). After another two weeks, his hand recovered very well (Fig. 1d).

**Table 1:** In vitro susceptibility of *E. jeanselmei* isolates to antifungal agents

Resource	Number of isolate (N)	Year of report	MIC (µg/mL)								
			AMB	FLC	ITC	VRC	POS	CAS	MFG	AFG	ISC
This Study	1	2021	1	ND	0.25	0.125	0.25	0.25	0.03	0.25	ND
[111]	14	2021	0.25–2	8–64	0.031–0.25	0.25–4	≤ 0.016–0.125	0.008–4	≤ 0.008–4	ND	0.25–4
[112]	6	2021	0.25–4	ND	0.03–0.25	0.06–0.25	0.03–0.25	0.125–2	0.125	0.094	ND
[59]	1	2019	2	16	0.5	0.5	ND	4	0.25	ND	ND
[113]	1	2016	1	ND	0.125	0.25	0.06	ND	ND	ND	ND
[81]	1	2013	0.25	32	0.125	0.25	< 0.03	ND	ND	ND	ND
[47]	9	2010	0.25–2	8–32	0.031–0.25	0.25–2	0.016–0.063	2–8	ND	0.063–4	0.25- > 2
[105]	2	2010	0.25–0.5	18–20	0.063–0.05	0.2–0.78	ND	ND	ND	ND	0.2–3.1
[50]	1	2010	0.5	> 64	0.06	ND	ND	2	ND	ND	ND
[103]	7	2010	0.5	ND	0.03	0.125	≤ 0.015	ND	ND	ND	ND
[114]	8	2009	0.25–1.0	ND	≤ 0.015–0.125	0.06–0.5	≤ 0.015–0.03	ND	ND	ND	ND
[63]	1	2008	0.5	ND	0.125	0.03	ND	ND	ND	ND	ND
[39]	1	2005	1	ND	64	ND	ND	ND	ND	ND	ND
[115]	5	2004	2–16	ND	ND	0.5–2	ND	ND	ND	0.125–2	ND
[38]	1	2004	0.25	ND	0.03	ND	ND	ND	ND	ND	ND
[72]	9	2001	0.25–0.5	ND	0.125–0.25	0.5–1	0.125–0.5	ND	ND	ND	ND
[90]	1	2000	0.5	ND	1	ND	ND	ND	ND	ND	ND

*AMB* amphotericin B, *FLC* fluconazole, *ITC* itraconazole, *VRC* voriconazole, *POS* posaconazole, *CAS* caspofungin, *MFG* micafungin, *AFG* anidulafungin, *ISC* isavuconazole, *ND* not determined, MIC (minimum inhibitor concentration)

## Literature Review of the Cases Associated with *E. jeanselmei*

A total of 89 cases associated with *E. jeanselmei* during 1980 to 2020 were collected from PubMed database (http://www.ncbi.nlm.nih.gov/pubmed), but five of them were excluded for lack of detailed data [2–4, 6, 8–92]. We summarized the available results of in vitro susceptibility testing against 70 strains of *E. jeanselmei* described in Table 1. Except one study described the E-test method [39], broth microdilution method were performed in the other reports. The antifungal agents tested frequently were amphotericin B, azoles and echinocandins. In addition, two studies tested the activity of terbinafine [38, 59].

The 84 cases included were classified into immunocompetent or immunocompromised group according to their underlying conditions. The clinical characteristics of the infections among these two groups were compared as shown in Table 2.

**Table 2:** Clinical characteristics of *E. jeanselmei*-related cases published in PubMed database during 1980 to 2020

Characteristics	Immunocompetent group (N = 29)	Immunocompromised group (N = 55)	χ2	*P* value
Age	56.1 ± 15.3	51.1 ± 23.8	0.234	0.629
*Sex*			0.624	0.429
Male	21	44		
Female	8	11		
*Location of infection*			2.096	0.351
Upper limbs	15	31		
Lower limbs	13	18		
Other	1	6		
*Treatment choice*			4.382	0.223
Surgery	0	3		
Antifungal agents	18	23		
Both	9	21		
NA	2	8		
*Clinical Outcome*			1.185	0.553
Survived	22	42		
Died	2	7		
NA	5	6		
*Length of treatment *(weeks)			1.937	0.586
≤ 8	4	12		
8–24	7	11		
≥ 24	11	13		
NA	7	9		

NA, not available

## Discussion

Phaeohyphomycosis is a rare infection caused by the dematiaceous fungi [93]. In recent years, the frequent occurrence of this kind of fungal infection as well as the diversity of the organisms isolated has been reported around the world [94, 95]. *Alternaria* spp. was associated with the cutaneous phaeohyphomycosis in solid-organ transplant recipients [89, 96, 97]. *Scedosporium prolificans* was a common species (41.6%) responsible for disseminated phaeohyphomycosis [98]. *E. jeanselmei* was involved in the subcutaneous or cutaneous infections [65, 99]. *Exophiala* spp. was isolated from 80.0% of the cases had underlying collagen disease and *E. jeanselmei* was the most common species (46.2%) [2]. In this study, we shared an experience of successful diagnosis and treatment of a prolonged cutaneous phaeohyphomycosis due to *E. jeanselmei,* and also reviewed the cases associated with this species.

Accurate identification of the pathogen can provide an important basis for precise diagnosis and treatment of phaeohyphomycosis. Otherwise, prolonged disease process, disseminated or relapsed situation, even poor clinical outcomes might be brought about [100, 101]. Traditionally, histopathological examination and culture-based methods are applied to identify the fungal pathogen. Whereas, these methods are usually time-consuming and confused morphological characteristics might bring the challenges for the inexperienced technicians. Therefore, molecular-based methods are preferred especially when typical morphological characteristics cannot be observed. In our case, the internal transcribed spacer (ITS) regions of the isolate were sequenced. Eventually, a cutaneous phaeohyphomycosis associated with *E. jeanselmei* was diagnosed based on the clinical features, histopathological examination, culture and ITS sequencing of the isolate.

In our study, in vitro susceptibility of *E. jeanselmei* isolates to antifungal agents were also reviewed. As shown in Table 1, this species had good in vitro susceptibility to amphotericin B, posaconazole, voriconazole, itraconazole and echinocandins. Variable activity with wide MIC range of amphotericin B (0.25–16 µg/mL), voriconazole (0.03–4 µg/mL) and itraconazole (< 0.015–64 µg/mL) were also observed in Table 1. In practice, susceptibility testing for each isolate should be performed to guide the precise treatment. In addition, other factors should be considered when the agents were chosen. Amphotericin B was a potent broad-spectrum antifungal drug for fungal infections, but it is not the best choice due to severe side effects [102]. Azoles were reported as the active drugs against *E. jeanselmei* [103].Though itraconazole showed significant activity against dematiaceous fungi, adverse effects and the lack of an intravenous formulation have reduced its use [99, 104]. Voriconazole was likely a good choice due to its broad activity, preferable side effect profile, and availability of an intravenous formulation [105]. Echinocandins had a variable in vitro activity and were not suggested by the guidelines of European Society for Clinical Microbiology and Infectious Diseases and European Confederation of Medical Mycology (ESCMID/ECMM) [102]. Terbinafine was also the treatment option for cutaneous and subcutaneous infection [38]. The lack of serious side effects and broad-spectrum in vitro antifungal activity help make terbinafine an alternative drug for the patients who cannot be cured through conventionally recommended treatments [106].

According to ESCMID/ECMM guidelines, antifungal therapy combination with surgical excision was recommended for managing phaeohyphomycosis [102]. In our review, we found that antifungal therapy (48.4%) and antifungal therapy combined with surgery (35.7%) were the common choices for treating the infections caused by *E. jeanselmei.* The surgical resection was recommended for treating the phaeohyphomycosis in immunocompromised host, because the recurrence of the infection occurred in about 30.0% of these patients [107]. The surgical drainage was suggested when the surgical excision was not applicable for the patients [4]. Modifying immunosuppression condition could improve the outcome of antifungal treatment in transplant patients [6, 96, 108]. In terms of the patients’ outcome, we found that 76.2% of cases recovered. Poor outcome was usually associated with the misdiagnosis, invalid treatment, or the occurrence of disseminated infections [109].

We also found that more than 80.0% of the cases occurred in the patients over 50 years old and 95.2% of the related infections presented as localized subcutaneous or cutaneous lesions. The trauma, particularly in the face and extremity (61.9%), might be the common cause of the infections, which could lead to the inoculation of the fungus in the cutaneous or subcutaneous tissue as described previously [93, 110].

In addition, we payed attention to the correlation between immunity status and clinical characteristics of the infections caused by *E. jeanselmei*. As listed in Table 2, there were no statistically significant for the location of infection, treatment choice, length of treatment and patient’s outcome between immunocompromised groups and immunocompetent groups.

In all, the results of histopathological examination, culture and species identification can provide the important basis for precise diagnosis and effective treatment of the infection due to *E. jeanselmei*, as demonstrated in our case. The findings of our review based on the cases reported in PubMed database indicated that *E. jeanselmei* isolates had good in vitro susceptibility to antifungal agents and immune status of the patients might not be correlated to the characteristics of the infections.
